# Term Newborns with relatively low Tissue Oxygen Saturation Levels soon after Birth are predisposed to Neonatal Respiratory Disorders in Low-risk, Elective Cesarean Sections

**DOI:** 10.7150/ijms.53945

**Published:** 2021-03-30

**Authors:** Kenta Kawai, Toshiyuki Uchida, Mari Mukai, Masako Matsumoto, Toshiya Itoh, Tomoaki Oda, Yoshimasa Horikoshi, Kazunao Suzuki, Yukiko Kohmura-Kobayashi, Naomi Furuta-Isomura, Chizuko Yaguchi, Masatsugu Niwayama, Hiroaki Itoh, Naohiro Kanayama

**Affiliations:** 1 Department of Obstetrics and Gynecology, Hamamatsu University School of Medicine, Hamamatsu, Japan.; 2 Department of Electrical and Electronics Engineering, Shizuoka University, Hamamatsu, Japan.; 3 Research Institute of Electronics, Shizuoka University, Hamamatsu, Japan.

**Keywords:** fetal tissue oximetry, fetal tissue oxygen saturation, near-infrared spectroscopy, parturition

## Abstract

**Background:** Neonatal respiratory disorders, such as transient tachypnea of the newborn and respiratory distress syndrome, occur frequently after an elective cesarean delivery. Although conventional pulse oximetry is recommended for neonatal resuscitation, it often requires several minutes after birth to obtain a reliable signal. In a previous study, we used novel tissue oximetry equipment to detect fetal and neonatal early tissue oxygen saturation (StO_2_) before and immediately after vaginal delivery. Therefore, we hypothesized that low neonatal StO_2_ levels measured by tissue oximetry may lead to neonatal respiratory disorder after a scheduled cesarean delivery. Hence, this study aimed to evaluate the StO_2_ levels measured by tissue oximetry in neonates with or without a respiratory disorder subsequently diagnosed after an elective cesarean delivery.

**Materials and methods:** We enrolled 78 pregnant Japanese women who underwent an elective cesarean section at ≥36 weeks' gestation. After combined spinal and epidural anesthesia were administered to the mother, fetal StO_2_ levels were measured by tissue oximetry using an examiner's finger-mounted sensor during a pelvic examination immediately before the cesarean section. We measured the neonatal StO_2_ levels at 1, 3, and 5 minutes after birth and retrospectively compared the fetal and neonatal StO_2_ levels with the incidence of subsequent diagnoses of neonatal respiratory disorders.

**Results:** The data of StO_2_ levels in 35 neonates were collected. Seven neonates (respiratory disorder (RD) group) were subsequently diagnosed with respiratory disorders by neonatal medicine specialists, whereas the 28 remaining neonates (NR group) were not. The median fetal StO_2_ (interquartile range) of the RD and NR groups was 52.0% (41.8%-60.8%) and 42.5% (39.0%-52.5%), respectively (*P* = 0.12). The median neonatal StO_2_ (interquartile range) of the RD and NR groups at 1 minute after birth was 42.0% (39.0%-44.0%) and 46.0% (42.0%-49.0%), respectively (*P* = 0.091). At 3 minutes after birth, the median neonatal StO_2_ (interquartile range) of the RD and NR groups was 41.0% (39.0%-46.0%) and 47.0% (44.3%-53.5%), respectively (*P* = 0.004). Finally, at 5 minutes after birth, the median neonatal StO_2_ (interquartile range) of the RD and NR groups was 45.0% (44.0%-52.0%) and 54.0% (49.3%-57.0%), respectively (*P* = 0.007).

**Conclusions:** The StO_2_ values in the RD group were lower than those in the NR group at 3 and 5 minutes after birth, suggesting that neonates with low StO_2_ levels soon after birth may be predisposed to clinically diagnosed neonatal respiratory disorders.

## Introduction

Compared with term vaginal delivery, elective cesarean delivery is a risk factor for neonatal respiratory disorders such as transient tachypnea of the newborn (TTN) and respiratory distress syndrome 1,2. Exposure to labor may initiate processes that enhance the resorption and clearance of alveolar fluid, thereby reducing the risk of neonatal respiratory disorders 3. In a retrospective cohort study of 29 669 deliveries, TTN occurred approximately 3 times more often after a scheduled cesarean delivery than after a vaginal delivery (3.1% vs. 1.1%) 1. However, diagnostic criteria or reliable tests on resuscitation involving neonatal respiratory disorders have not been completely established, especially given the high incidence of neonatal respiratory disorders after elective cesarean delivery 4. Although TTN is a common disorder, it is diagnosed clinically by exclusion; in clinical studies, the “diagnostic criteria of transient tachypnea of the newborn included tachypnea and imaging studies characterized by nonspecific signs” 5.

The 2015 American Heart Association/American Academy of Pediatrics/International Liaison Committee on Resuscitation (AHA/AAP/ILCOR) guidelines recommend the use of pulse oximetry for measuring oxygen saturation (SpO_2_) during resuscitation because neonatal appearance and skin color (i.e., cyanosis) are poor indicators of neonatal hypoxia 6,7. However, pulse oximetry does not provide an accurate signal immediately after birth 7-10. The failure of pulse oximetry to obtain blood oxygen saturation (SpO_2_) measurements within 5 minutes of life has been pointed out 8,9. Moreover, van Vonderen 11 reported that during the first 2 minutes after birth, pulse oximetry frequently displays a newborn heart rate that differs from that obtained by a simultaneous electrocardiogram.

We recently developed a noninvasive fetal and neonatal tissue oximetry system that uses an examiner's finger-mounted near-infrared spectroscopy sensor (NIRS) (KN-15, ASTEM Co., Ltd., Kawasaki, Japan). This 1 cm sensor provides immediate tissue oxygen saturation (StO_2_) data when an examiner touches the probe to an arbitrary site 12. This novel device has been validated across various settings, such as animal experiments 13,14, fetal measurements 15,16, neonatal measurements 16,17, and limb measurements 18. Uchida et al. 15 reported that fetal StO_2_ in the second stage of term labor significantly correlated with umbilical arterial pH analyzed after delivery. Moreover, Mukai et al. 16 reported a longitudinal assessment of StO_2_ using this novel device from the fetal to neonatal periods during vaginal delivery. Watanabe et al. 17 compared the neonatal StO_2_ measurements using this novel device with the SpO_2_ measurements obtained by pulse oximetry and found that the novel device provided a more stable measurement than pulse oximetry did.

In the present study, we hypothesized that fetal and neonatal StO_2_ levels measured by tissue oximetry using an examiner's finger-mounted NIRS sensor are low among neonates diagnosed with respiratory disorders after low-risk, term cesarean deliveries. This study specifically aimed to longitudinally measure fetal and neonatal StO_2_ levels (immediately before delivery and 1, 3, and 5 minutes after birth) in low-risk, term elective cesarean sections.

## Materials and Methods

### Participants

We conducted a nested case-control study and recruited pregnant women who underwent elective cesarean sections in the Perinatal and Neonatal Care Center in the Hamamatsu University Hospital from May 2016 to March 2018. We included elective cesarean deliveries performed ≥ 36 weeks, 0 days of gestation. The indications for an elective cesarean section included a previous uterine operation, breech presentation, or twin pregnancy. Conversely, the exclusion criteria were as follows: 1) rupture of membranes before cesarean section, 2) labor prior to cesarean section, 3) intact cervical os, 4) placenta previa detected by vaginal ultrasound, 5) twin pregnancy, 6) fetal malformations, or 7) nonreassuring fetal status or maternal complications before delivery.

### Instruments

**Figure 1:**
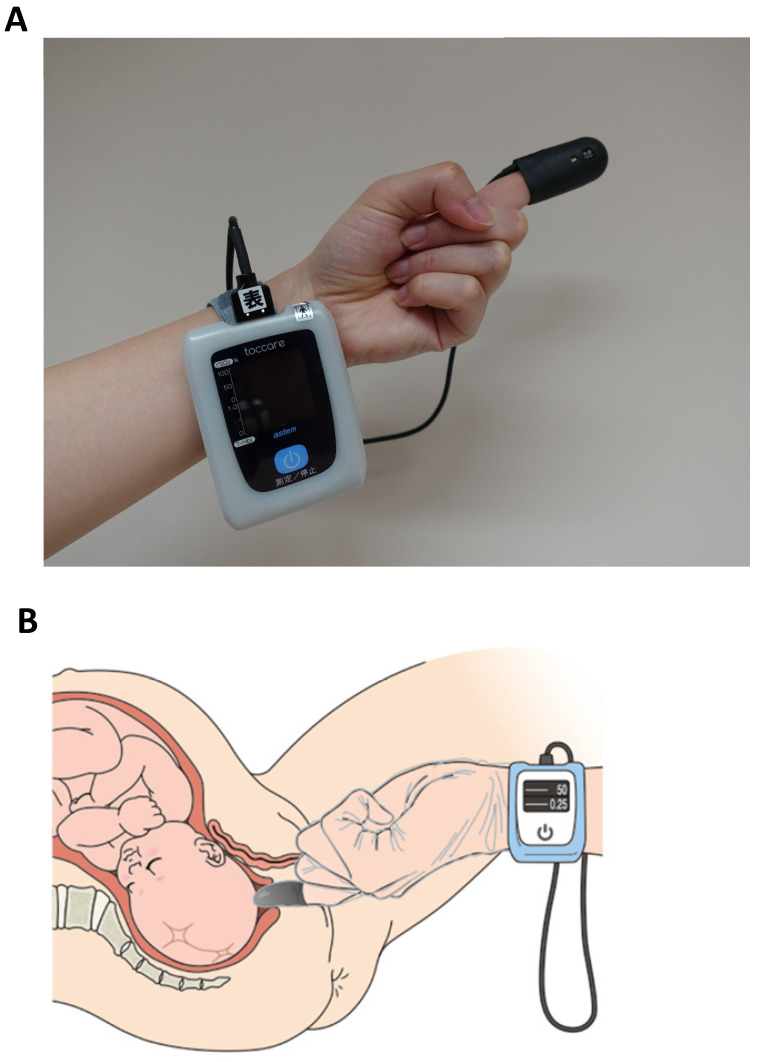
Tissue oximetry probe mounted on an examiner's finger (panel A). The examiner touches the probe to the fetus during a pelvic examination to measure the fetal tissue oxygen saturation levels (panel B). Panel B is cited by 27 Uchida T, Kanayama N, Kawai K, et al. Reevaluation of intrapartum fetal monitoring using fetal oximetry: A review. *J Obstet Gynaecol Res*. 2018;44(12):2127-2134. Copyright © 2018 Japan Society of Obstetrics and Gynecology. Re-use permitted.

We utilized a novel tissue oximetry system that measures regional StO_2_ levels noninvasively using the spatially resolved NIRS. The wavelengths of the light from the light-emitting diodes (LEDs) were 770 and 830 nm (KN-15, ASTEM Co., Ltd., Kawasaki, Japan) (Figure 1A). A probe containing a sensor was mounted on an examiner's finger and covered by a sterile polyethylene glove (Singer disposable gloves, Utsunomiya Seisaku Co., Ltd., Osaka, Japan) to protect against infection. Light detectors were located 6 and 8 mm from the LEDs. We determined the oxyhemoglobin, deoxyhemoglobin, and StO_2_ values by using the spatially resolved NIRS 12. The novel tissue oximetry device enabled us to noninvasively and immediately measure regional StO_2_ levels when the examiner touched the finger-mounted sensor to a target region, such as the fetal and neonatal head 15-17.

### Measurement protocol

Fetal and neonatal StO_2_ levels were measured for at least 5 seconds until the values stabilized. The values were measured after the combined spinal and epidural anesthesia was administered to the mother before the cesarean section (Figure 1B) and at 1, 3, and 5 minutes after delivery 15-17. Because the fetal StO_2_ levels were measured during an elective cesarean section before the onset of labor, some cervical canals were still immature and partially opened. Therefore, probes mounted on the examiner's finger might have touched the maternal tissues (StO_2_ levels around 60%) instead of the fetal tissues (around 40%). Therefore, we measured the StO_2_ levels of the uterine cervix separately and excluded the subjects if the StO_2_ levels were more than 55% by analyzing the receiver operating character (ROC) curve (Supplementary material). All the subjects were measured by a maternal-fetal medicine specialist (KK). Routine neonatal care was given according to the 2015 AHA/AAP/ILCOR guidelines. Neonates who were clinically determined by neonatal specialists as needing any respiratory support on admission were retrospectively classified as belonging to the group with neonatal respiratory orders (the RD group), whereas the remaining neonates were classified as belonging to the NR group.

### Statistics

The ratio scale values were expressed as median and interquartile ranges. The nominal scale values were expressed as numbers and percentages. Significant differences between 2 values were assessed using the Welch *t*-test or the Fisher exact test. Chronological changes of the values were examined using the Friedman test with the Wilcoxon signed-rank test for post hoc analysis adjusted with the Holm method. The cutoff values for StO_2_ were obtained by calculating the maximal Youden's J statistic in ROC analysis. The intra-rater and inter-rater reliability values were expressed using the intraclass correlation coefficient (ICC). Statistical analysis was performed using the statistical software R version 3.4.1 (R Core Team [2017]; R: A language and environment for statistical computing; R Foundation for Statistical Computing, Vienna, Austria. URL https://www.R-project.org/) and JMP version 13.2.1 (SAS Institute Inc., Cary, NC, USA).

### Ethical considerations

This research related to human use complied with all the relevant national regulations and institutional policies, and in accordance with the tenets of the Helsinki Declaration, was approved by the authors' institutional review board. The Ethics Committee of the Hamamatsu University School of Medicine (Registration number 19-048) approved this study, and all participants provided written informed consent.

## Results

**Figure 2:**
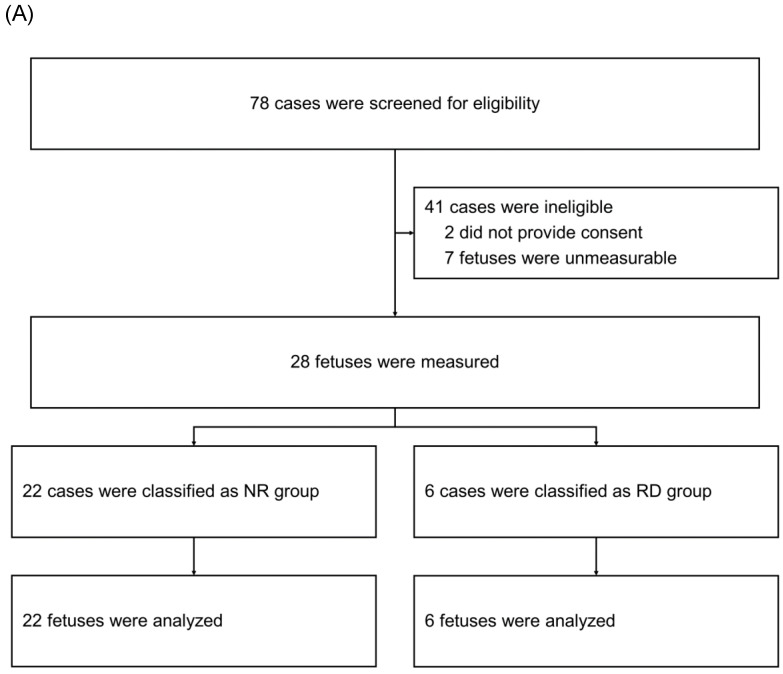

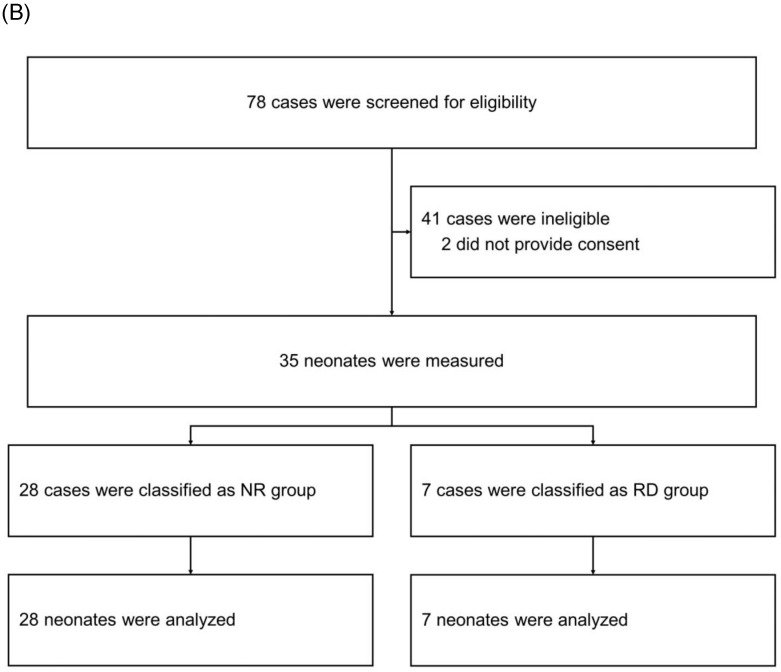
Enrollment and classification of fetuses (panel A) and neonates (panel B). NR Group: fetuses and neonates who were not subsequently diagnosed with respiratory disorders; RD Group: fetuses and neonates who were subsequently diagnosed with respiratory disorders.

**Figure 3:**
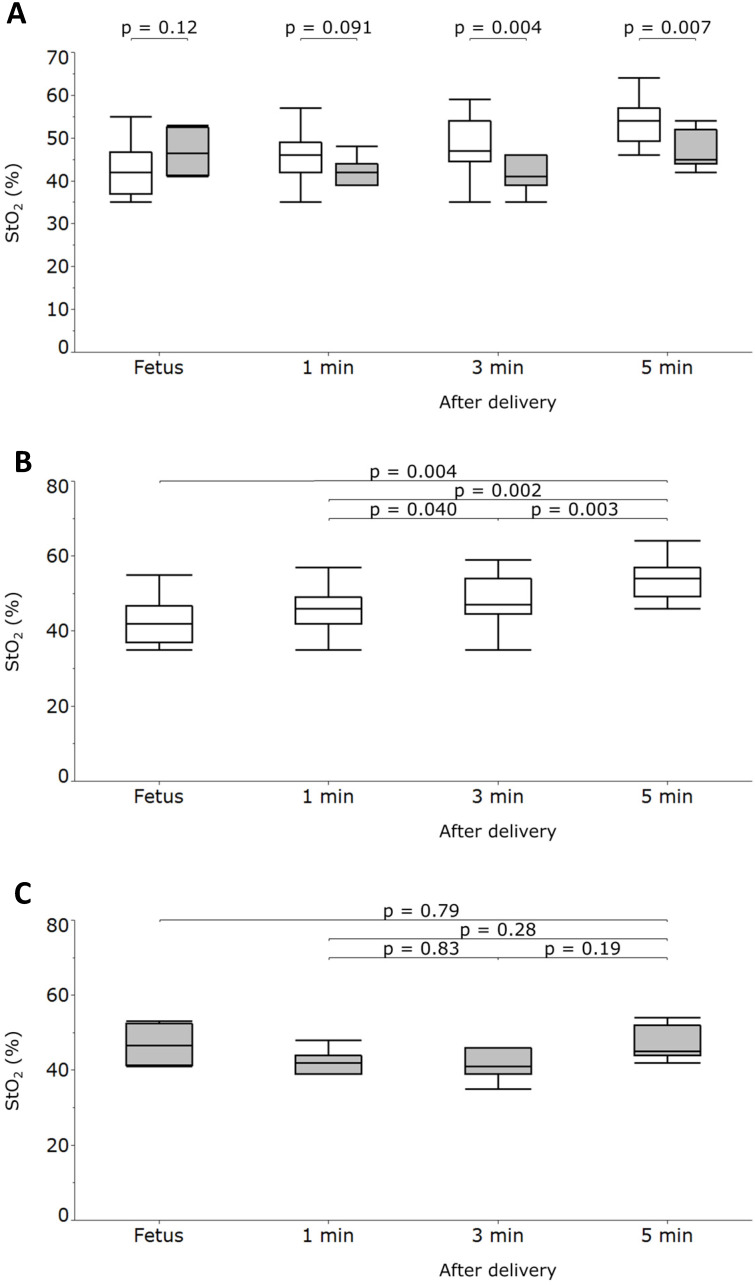
Tissue oxygen saturation (StO_2_) values measured in fetuses and neonates who were subsequently diagnosed with or without respiratory disorders (RD and NR groups, respectively) at 1, 3, and 5 minutes after delivery. Fetal StO_2_ values were not significantly different between the 2 groups. At 3 and 5 minutes after delivery, the NR group had significantly higher StO_2_ values than the RD group (panel A). StO_2_ values measured in the NR group increased significantly (panel B), whereas those in the RD group did not change significantly (panel C). Box plots represent the lowest datum that is still within 1.5 interquartile range (IQR) of the lower quartile, the lower quartile, the median, and the upper quartile; the highest datum was still within 1.5 IQR of the upper quartile. Gray boxes represent the RD group, and the white boxes represent the NR group. *P* values were calculated using the Welch *t*-test or the Friedman test, and the Wilcoxon signed-rank test for post hoc analysis adjusted with the Holm method.

After screening 78 mothers for eligibility, we subsequently enrolled 35 mothers (Figure 2). Among the 35 neonatal cases, 7 were retrospectively classified as having a neonatal respiratory disorder (RD group), whereas the remaining 28 were categorized as having no such disorder (NR group). We compared the fetal and neonatal StO_2_ of both groups (Table 1, Figure 3A). Neonatal StO_2_ values were obtained in all 35 neonates; however, fetal StO_2_ values were only obtained in 28 neonates (RD group, n = 6; NR group, n = 22). Perinatal background, such as maternal age, gestational weeks, birth weight, umbilical arterial pH, and Apgar scores, were similar between the RD and NR groups. Notably, 85.7% of neonates in the RD group required resuscitation, including continuous positive airway pressure, positive pressure ventilation, oxygen supplementation, and/or intubation, but only 17.9% of those in the NR group required resuscitation (Table 1).

There were no statistically significant differences in fetal StO_2_ values or neonatal StO_2_ values at 1 minute after birth between the two groups (Table 1, Figure 3A).

**Figure 4:**
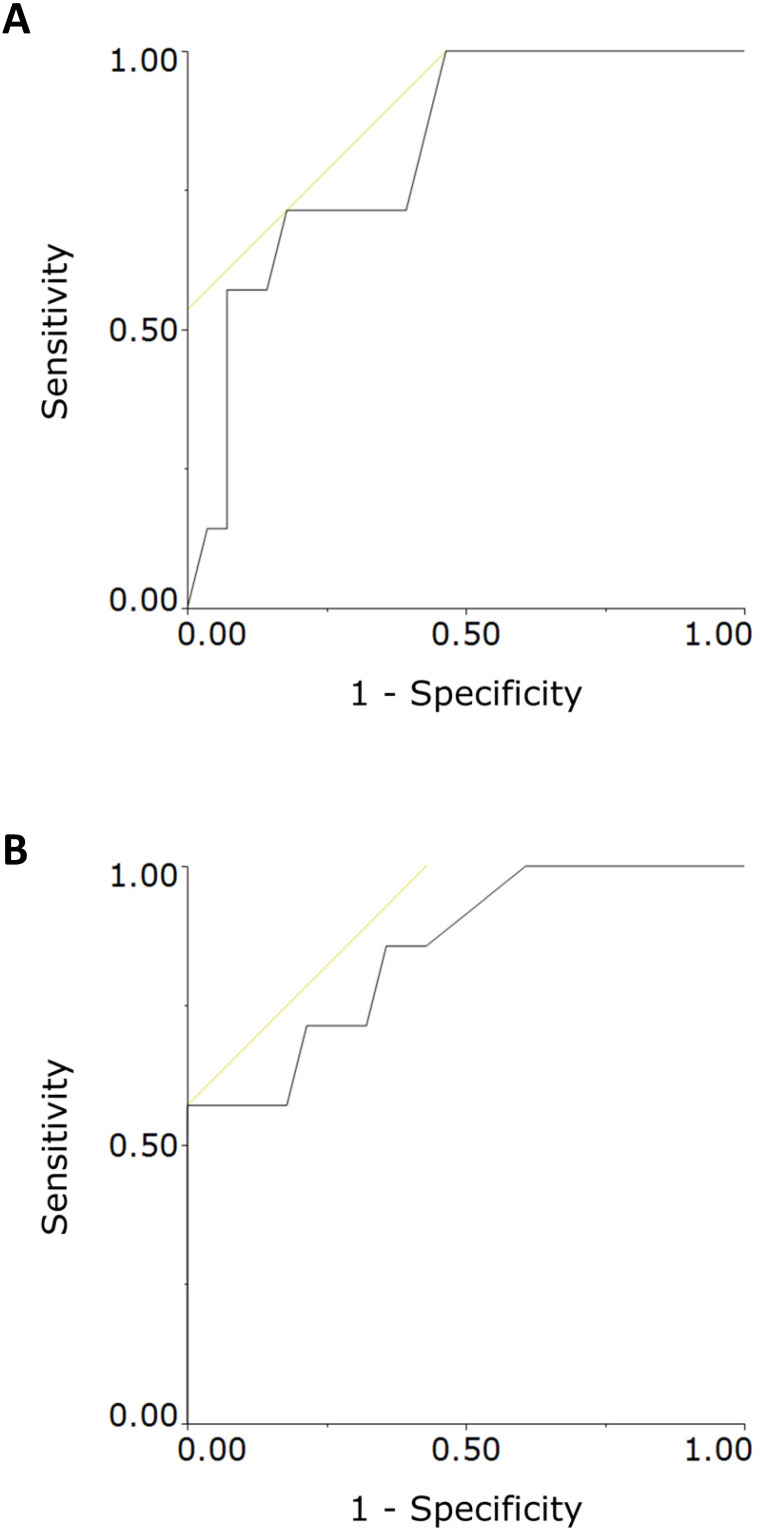
Receiver operating characteristic (ROC) curve analysis of neonatal tissue oxygen saturation (StO_2_) values measured at 3 minutes (panel A) and 5 minutes (panel B) after birth. The cutoff values of 43% and 45% for distinguishing neonates who were and were not subsequently diagnosed with respiratory disorders were obtained by calculating the maximal Youden's J statistic in ROC curve analysis. The areas under the curve were 0.821 and 0.849 for the 43% and 45% cutoff values, respectively.

The longitudinal changes of the NR group's neonatal StO_2_ values were similar to those observed in our previous report 16,17. However, the RD group had significantly lower neonatal StO_2_ values at 3 and 5 minutes after birth (Table 1, Figure 3A). Moreover, the NR group's neonatal StO_2_ values increased chronologically, whereas those of the RD group did not (Figure 3B, 3C). For the RD group, ROC analysis showed the cutoff values of StO_2_ at 3 and 5 minutes after delivery were 43%, and 45%, respectively (Figure 4). The intra-rater reliability for neonatal StO_2_ using the ICC (1, 1) and (1, 5) was 0.88 and 0.97, respectively, whereas the inter-rater reliability for neonatal StO_2_ using the ICC (3, 1) was 0.71.

## Discussion

Although we could not measure StO_2_ in some fetuses because of unfavorable cervical maturation, we successfully measured StO_2_ in all neonates at 1, 3, and 5 minutes after birth, indicating StO_2_ is a stable and reliable way to assess neonatal oxygenation soon after delivery. This study demonstrated that newborns who were clinically diagnosed with subsequent neonatal respiratory disorders had significantly lower neonatal StO_2_ values at 3 and 5 minutes after birth compared with those newborns without respiratory disorders (Figure 3). Moreover, the StO_2_ values before and after cesarean delivery did not increase chronologically in neonates with respiratory disorders (Figure 3). Additionally, there were no significant differences in fetal StO_2_ values before the elective cesarean sections between neonates with or without subsequently diagnosed neonatal respiratory disorders.

The 2015 AHA/AAP/ILCOR guidelines recommend the use of conventional pulse oximetry during resuscitation; however, pulse oximetry takes several minutes to obtain a reliable signal, especially immediately after birth 7-10. Therefore, this method is not considered diagnostically useful because it remains unclear whether neonatal oxygenation levels during the first several minutes after birth as measured by pulse oximetry are reliable. Watanabe et al. 17 demonstrated that reliable StO_2_ levels measured by tissue oximetry (KN-15) could be obtained earlier than SpO_2_ levels measured by conventional pulse oximetry. We recently reported the ability to measure neonatal StO_2_ at 5 minutes after vaginal delivery 16, but in the present study, we successfully measured neonatal StO_2_ in all cases at 1, 3, and 5 minutes after cesarean birth. In the previous studies using tissue oximetry (KN-15), the mean or median value of StO_2_ at 5 minutes after vaginal delivery was approximately 55% 16,17, which was similar to the median value of 54.0% at 5 minutes after cesarean delivery in the current study's NR group. Meanwhile, the reference ranges for neonatal SpO_2_ in cesarean births were lower than those in vaginal births 9,19,20. The reference value for SpO_2_ at 5 minutes after cesarean birth was reportedly 85% (60%-94%) 20. Similar devices using NIRS have also been used for measuring neonatal oxygenation immediately after birth; however, not all the values obtained by such devices have been reliable 21-23. Median values for the cerebral tissue oxygen saturation index (NIRO-200NX, Hamamatsu Photonics K.K., Hamamatsu, Japan) and for the regional cerebral tissue oxygen saturation (INVOS 5100, Somanetics Corp, Troy, MI, USA) were 65.6% and 66.8%, respectively. These values were higher than the StO_2_ values 22,23.

In this study, the neonatal StO_2_ values measured at 3 and 5 minutes after birth were significantly lower in neonates clinically diagnosed with subsequent neonatal respiratory disorders than in those without respiratory disorders. As mentioned, elective cesarean delivery at term is a risk factor for developing neonatal respiratory disorders such as TTN and respiratory distress syndrome as compared with vaginal delivery at term 1,2. However, TTN has had no established criteria for diagnosis and continues to be diagnosed multilaterally according to clinical findings such as tachypnea and respiratory distress and imaging findings such as chest radiograph and ultrasonography 4,5,24,25. In a previous study, there were no significant differences in neonatal SpO_2_ values at 10 minutes after birth between neonates with TTN and those in a control group 26. The present findings suggest that the cutoff values of 43% (3 minutes), and/or 45% (5 minutes) might be useful in identifying newborns at high risk for developing subsequent neonatal respiratory disorders after cesarean delivery (Figure 4). A large multicenter study is necessary to reconfirm the cutoff values. The present findings suggest that the assessment of StO_2_ within 5 minutes after birth might provide guidance to help improve neonatal resuscitation, especially after an elective cesarean section. We have no clear explanation as to why fetal StO_2_ values in the RD group were higher than those in the NR group.

The present study, however, has some limitations. First, neither the neonatal SpO_2_ nor the partial pressure of arterial oxygen was available because the subjects were scheduled for low-risk elective cesarean sections. Second, some newborns in the RD group required resuscitation, including supplemental oxygen and positive pressure ventilation, while fewer newborns in the NR group required resuscitation, thereby possibly affecting the difference in StO_2_ levels. The StO_2_ values were measured independently by the author and concealed from the clinicians. Third, we only enrolled cases of elective cesarean section; therefore, further studies are necessary in cases of emergency cesarean section and vaginal delivery. Moreover, the present subjects underwent elective cesarean section without any clinical findings of nonreassuring fetal status. Our research group is conducting an ongoing study of the predictive value of low StO_2_ before an emergency cesarean section in cases of nonreassuring fetal status.

In conclusion, neonatal StO_2_ data were obtained in all cases after scheduled cesarean delivery. Neonatal StO_2_ values measured at 3 and 5 minutes after birth were significantly lower in neonates who were subsequently diagnosed with neonatal respiratory disorders than in those who were not, suggesting that neonates with low StO_2_ levels soon after birth might be predisposed to subsequent clinically diagnosed neonatal respiratory disorders.

## Supplementary Material

Supplementary materials and figures.

## Figures and Tables

**Table 1:** Comparison of the baseline characteristics and measured data of fetuses and neonates who were subsequently diagnosed with a respiratory disorder (RD group) and those who were not (NR group)

	NR Group (n = 28)	RD Group (n = 7)	*P* value
*A. Characteristics*			
Maternal age, years	35.0 (33.0-37.0)	32.0 (26.0-40.0)	0.52
Gestational age, weeks	37.9 (37.6-38.1)	38.0 (37.7-38.3)	0.33
Birth weight, g	2878 (2725-3177)	2854 (2818-3068)	0.95
Umbilical arterial pH	7.308 (7.282-7.334)	7.332 (7.309-7.336)	0.15
Apgar score at 1 min	8.0 (8.0-8.0)	8.0 (8.0-8.0)	0.84
Apgar score at 5 min	9.0 (9.0-9.0)	9.0 (8.0-9.0)	0.61
Cervical dilation, cm	2.0 (1.0-2.5)	1.5 (1.0-2.0)	0.66
Neonatal resuscitation, n	5 (17.9)	6 (85.7)	0.002
Cephalic presentation, n	20 (71.4)	6 (85.7)	0.65
*B. Measured data*			
StO_2_ of fetus, %	42.5 (39.0-52.5)^a^	52.0 (41.8-60.8)^b^	0.12
StO_2_ at 1 min after delivery, %	46.0 (42.0-49.0)	42.0 (39.0-44.0)	0.091
StO_2_ at 3 min after delivery, %	47.0 (44.3-53.5)	41.0 (39.0-46.0)	0.004
StO_2_ at 5 min after delivery, %	54.0 (49.3-57.0)	45.0 (44.0-52.0)	0.007

Values are presented as median with interquartile range in parentheses, except for cephalic presentation, which shows n with percentage in parentheses. *P* values were calculated with the Welch *t*-test for all variables, except for cephalic presentation, where the Fisher exact test was used, ^a^n = 22, ^b^n = 6. NR Group: fetuses and neonates who were not subsequently diagnosed with respiratory disorders; RD Group: fetuses and neonates who were subsequently diagnosed with respiratory disorders; StO_2_: tissue oxygen saturation. Neonatal resuscitation includes continuous positive airway pressure, positive pressure ventilation, oxygen supplementation, and/or intubation.
