# Supplementary material for: Term Newborns with relatively low Tissue Oxygen Saturation Levels soon after Birth are predisposed to Neonatal Respiratory Disorders in Low-risk, Elective Cesarean Sections
Source: Int J Med Sci. 2021 Mar 30;18(11):2262–8. doi: 10.7150/ijms.53945 (PMC8100654; doi:10.7150/ijms.53945)

Supplementary material

Tissue oxygen saturation (StO_2) values measured at the fetal site (Fetus) and the posterior lip of the uterine cervix (Cervix) during pelvic examination. The median values (interquartile range, IQR) of StO_2 measured at Fetus and Cervix were 44.0% (40.8%–53.3%), and 63.0% (59.3%–65.0%), respectively (panel A). StO_2 values measured at Cervix were significantly higher than those measured at Fetus ($P < 0.001$). The cutoff value of 55% for distinguishing Fetus with Cervix was obtained by calculating the maximal Youden’s J statistic in receiver operating characteristic curve analysis. The area under the curve was 0.927 (panel B). Box plots show the lowest datum that is still within 1.5 IQR of the lower quartile, the lower quartile, the median, and the upper quartile; the highest datum is still within 1.5 IQR of the upper quartile. The gray box represents the StO_2 values measured at Cervix, whereas the white box represents the StO_2 values measured at Fetus. The *P* value was calculated using the Welch *t*-test.

(A)

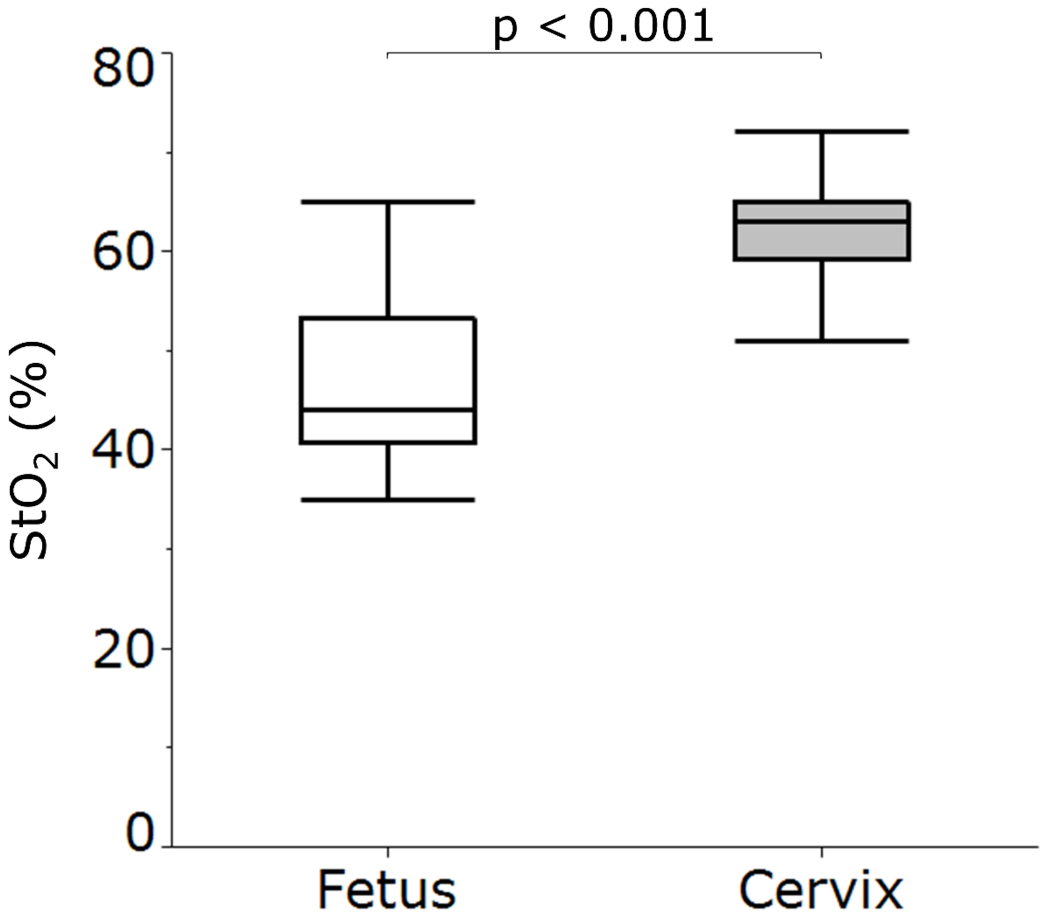

(B)

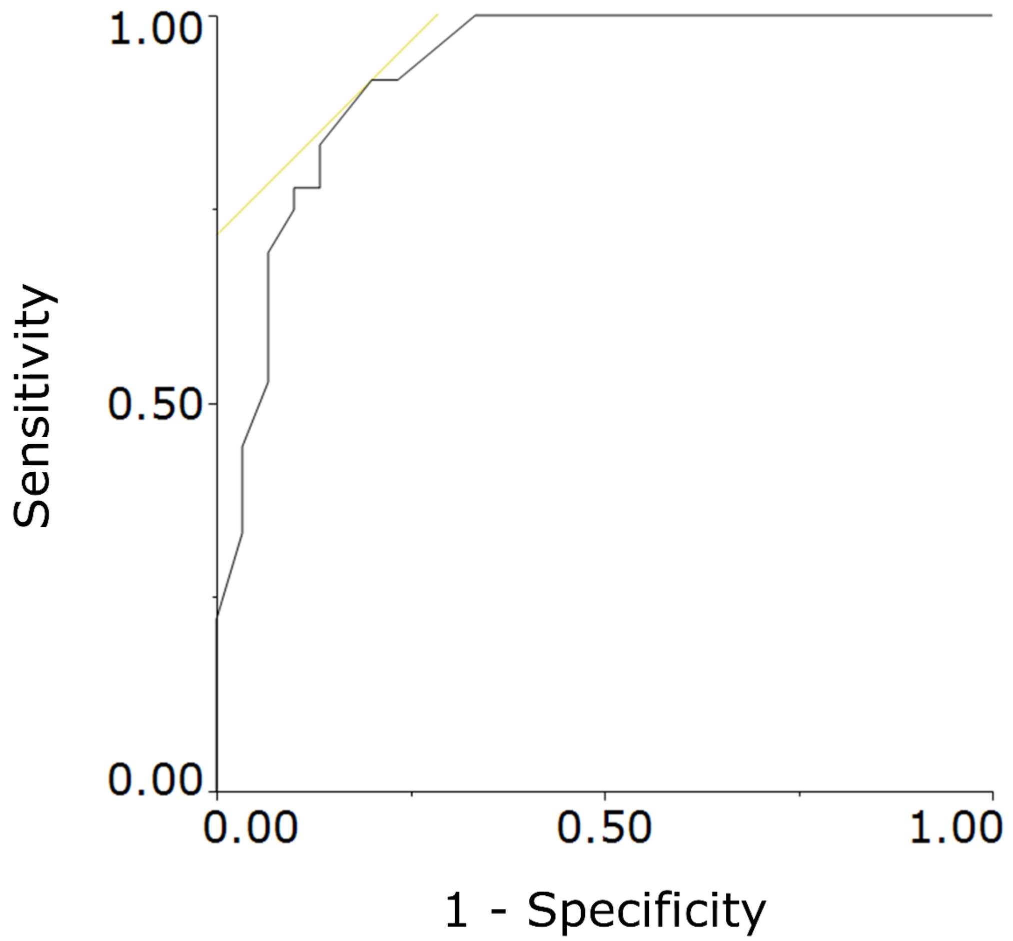

Supplement: Supplementary file 1 — Supplementary materials and figures. [file ijmsv18p2262s1.pdf]
